# Histological Stains: A Literature Review and Case Study

**DOI:** 10.5539/gjhs.v8n3p72

**Published:** 2015-06-25

**Authors:** Hani A Alturkistani, Faris M Tashkandi, Zuhair M Mohammedsaleh

**Affiliations:** 1 Faculty of Medicine, King Abdulaziz University, Jeddah, Saudi Arabia; 2 Faculty of Applied Medical Sciences, University of Tabuk, Tabuk, Saudi Arabia

**Keywords:** histological staining, histology, histopathology, histochemistry

## Abstract

The history of histology indicates that there have been significant changes in the techniques used for histological staining through chemical, molecular biology assays and immunological techniques, collectively referred to as histochemistry. Early histologists used the readily available chemicals to prepare tissues for microscopic studies; these laboratory chemicals were potassium dichromate, alcohol and the mercuric chloride to harden cellular tissues. Staining techniques used were carmine, silver nitrate, Giemsa, Trichrome Stains, Gram Stain and Hematoxylin among others.

The purpose of this research was to assess past and current literature reviews, as well as case studies, with the aim of informing ways in which histological stains have been improved in the modern age. Results from the literature review has indicated that there has been an improvement in histopathology and histotechnology in stains used. There has been a rising need for efficient, accurate and less complex staining procedures. Many stain procedures are still in use today, and many others have been replaced with new immunostaining, molecular, non-culture and other advanced staining techniques. Some staining methods have been abandoned because the chemicals required have been medically proven to be toxic. The case studies indicated that in modern histology a combination of different stain techniques are used to enhance the effectiveness of the staining process. Currently, improved histological stains, have been modified and combined with other stains to improve their effectiveness.

## 1. Introduction

Histology is the microscopic study of animal and plant cell and tissues through staining and sectioning and examining them under a microscope (electron or light microscope). There are various methods used to study tissue characteristics and microscopic structures of the cells. Histological studies are used in forensic investigations, autopsy, diagnosis and in education. In addition, histology is used extensively in medicine especially in the study of diseased tissues to aid treatment (Black, 2012).

Histological staining is a series of technique processes undertaken in the preparation of sample tissues by staining using histological stains to aid in the microscope study (Anderson, 2011). The process of histological staining takes five key stages which involve; fixation, processing, embedding, sectioning and staining (Titford, 2009). Great changes have been done on techniques used for histological staining through chemical, molecular biology assays and immunological techniques collectively and have facilitated greatly in the study of organs and tissues (Shostak, 2013).

## 2. Specific Aspects of Histopathology

### 2.1 Staining

Staining is used to highlight important features of the tissue as well as to enhance the tissue contrast. Hematoxylin is a basic dye that is commonly used in this process and stains the nuclei giving it a bluish color while eosin (another stain dye used in histology) stains the cell's nucleus giving it a pinkish stain. However, there are other several staining technicques used for particular cells and components (Black, 2012). Staining is a commonly used medical process in the medical diagnosis of tumors in which a dye color is applied on the posterior and anterior border of the sample tissues to locate the diseased or tumorous cells or other pathological cells (Musumeci, 2014). In biological studies staining is used to mark cells and to flag nucleic acids, proteins or the gel electrophoresis to aid in the microscopic examination (Jackson & Blythe, 2013). In some cases, various multiple staining methods are used such as differential staining, double staining or the multiple staining (Iyiola & Avwioro, 2011).

### 2.2 Fixation

In histology, fixation refers to the use of chemicals to preserve the natural tissue structure and maintain the cell structure from degradation. Mostly, neutral buffered formalin is used in this case when a light microscope is to be used to conduct the study. Fixatives enhance the preservation of tissues and cells through an irreversible process through cross-linking proteins. However, while the process serves to preserve the structure of the cell for the purpose of histological studies, it has been found to destroy and denature proteins rendering them dysfunctional (Young, O’Dowd, & Stewart, 2010). Formalin fixation denatures the DNA, miRNA and the mRNA tissues and extraction of these components for the purpose of histology may lead to flawed results (Anderson, 2011).

The fixation phase retains the chemical composition of the tissues, hardens the cells or tissues for sectioning and delays degradation (Titford, 2009). In addition, fixatives changes tissue penetration and influence antigen exposures which may be productive or detrimental (Iyiola & Avwioro, 2011). These fixatives are administered in two ways: through perfusion and immersion of the prepared tissue. These fixatives are infused in the animals’ body through diffusion. Perfusion is a slower process, require more time and only one fixative can be used at a time (Shostak, 2013). There are a number of fixatives in use, but the formaldehyde fixatives are the most commonly used (Black, 2012). The neutral buffered formalin (NBF) stabilizes amino acids in proteins and offers good tissues and cell structure preservation. The paraffin-formalin (paraformaldehyde- PFA) is effective in immunostaining but requires it to be freshly prepared to enhance its effectiveness (Iyiola & Avwioro, 2011). The Bouin fixative has been found to be effective in delicate and soft tissues such as small tissues, embryo and brain tissues (Musumeci, 2014). Bouin fixative offers good preservation of nuclei and the glycogen, but its penetrations are slow and distorts mitochondria and the kidney tissues (Weiss, Delcour, Meyer, & Klopfleisch, 2010).

**Dehydration:** In this step, the aim is to remove water from the selected tissues to solidify them and facilitate the cutting of thin sections of slides, more thinly for use in light microscopes and thick for the electron microscope. Water is removed from the tissues through the dehydration method through ethanol (Shostak, 2013). The process is repeated through a hydrophobic clearing substance such as xylene to remove the alcohol and paraffin wax and the infiltrated agent. Resins are used to enhance cutting of thin sections of the tissues (Titford, 2009).

**Embedding:** In staining, the process of embedding is done using paraffin wax to enhance easier extraction of cellular structures. In complex cellular tissues, plastic resin or wax is used, or combinations of fixatives are used to produce good morphology (Musumeci, 2014). However, these fixatives may lead to degradation of the cell and tissue structures due to prolonged heating, and this may lead to problems when conducting the hybridization process arising from the unstable RNA. In the same line, the infiltration of paraffin wax leads to inhibition of the penetration of antibody, chemical other fixatives. In order to alleviate this problem, freezing of tissues after the embedding, removing wax after staining and the use of PFA fixatives offers a reliable solution to improved morphology (Titford, 2009).

**Sectioning:** In histology sectioning refers to the preparation of ‘ribbon’ like microtomes of a tissue for the purpose of mounting it on a microscope slide for examination (Cai, Caswell, & Prescott, 2014). In this case, a series of thin sections of tissues of required thickness are cut and prepared through the paraffin method.

**Antigens Retrieval:** This is the next process after fixation and embedding and focuses on retrieving antigens that have been masked. When formalin fixatives are used as well as other aldehyde fixations the cross-linking of proteins leads to masking of the antigen sites, and this leads to weaker immunohistochemical staining. The antigen retrieval process serves to break protein cross-links and unmask the epitopes and the antigens that were fixed and embedded using formalin and paraffin (Titford, 2009). The overall strategy is to improve on the staining intensity of the antibodies (Cai, Caswell, & Prescott, 2014).

The commonly used antigen retrieval techniques are through heat-induced and proteolytic retrieval methods. The proteolysis digestion process should take the minimal dosage and time possible to avoid over digestion that may denature the tissue structures and the epitopes (Musumeci, 2014). The heat method leads to protein denaturalization and in some cases antigens are lost (Black, 2012). Similarly, heating may lead to the reversal of the chemical modifications induced during the fixation period. Heating from such devices as microwaves leads to chemical reactions of the protein structure (Shostak, 2013). However, a combination of enzymatic and heat retrieval methods lead to effective staining intensity (Godwin, 2011).

### 2.3 Gross and Microscopic Examination

The gross examination is a laboratory procedure in which pathological and medical examination is done through visible aspects of the eye. In microscopic examinations, pathological changes are done using a microscope (light or electronic microscope) (Musumeci, 2014). In most aspects, gross examination precedes microscopic examination in the identification of samples for microscopic examination. For instance, gross examination helps the pathologist identify the cells or tissues that have lumps (possibly cancer) but microscopic examination is used to confirm.

### 2.4 Some Advanced Histological Techniques

In the modern age of histology there have been significant improvements in histological stains and techniques. Advanced histological techniques are immunohistochemistry, antibody binding and electron microscopy (Titford, 2009). In the same line, advanced stains include: immunohistochemical (IHC), routine hematoxylin eosin (H&E) and the *in situ* hybridization (Musumeci, 2014). Modern stains used are;

- Masson's Stain used in connective tissues
- Golgi Stain used in neuronal fibresnnnn
- Toluidine Blue
- Immunological labeling that have fluorescent or enzymatic stains
- Kluver-Barrera Stain used in Lipofuscin
- Mallory's CT Stainssss
- Periodic Acid-Schiff (PAS) Stain used in carbohydrates

### 2.5 Regulations of Histologic in Different Countries

Most countries have standards and organizations that collaborate with national and international groups involved in the control and standardization of biological staining methods. Standardization is important in setting uniform criteria, methods and technical specifications of the stains used. The objective is to enhance establishment of procedures that produce stain substances that produce microscopic results capable of been reproducible in different countries in areas of cytology, bacteriology, histopathology and hematology (Lyon & Horobin, 2010).

Formal regulatory bodies that standardize stains and are independent of manufacturers are: International Organization for Standardization (ISO), European Committee for Standardization (CEN) and the American National Standards Institute (ANSI). Other bodies involved in the standardization of staining substances are: the USA Clinical Laboratory Standards Institute (CLSI), the World Health Organization (WHO) and the European Diagnostic Manufacturers Association (EDMA) among others. These regulatory bodies accredit, evaluate and approve manufacture and the use of staining dyes, antibodies, fluorochromes and the nucleic acid probes (Lyon & Horobin, 2010).

### 2.6 Objectives of the Study

A background study on commonly used histological staining techniques and stains indicate that some fixatives and techniques used in the histological processes are effective. However, some stains and processes are ineffective, and this leads to denaturalizationof tissues and cells which inhibit effective histological studies. The objective of this research was to assess past and current literature reviews and cases in the aim of informing ways in which histological stains have been improved in the modern histopathology. As a result, this study focuses on conducting an extensive and qualitative case study of past and present histological processes with the aim of understanding how histological strains could be improved.

## 3. Methodology

The research used an extensive exploration and review of historical, recent and current medical research studies and case studies in order to collect quantitative and qualitative data in regard to histological stains used in the past and recent cases (Silverman, 2011). In this case, a database of clinical pathology journals involving past and recent usage of histological stains was made. The identified pathological journals, articles, and case studies were reviewed, analyzed, and important trends in the use of histological stains were made. As such, through integrative and intensive literature and case study reviews rich, data were collected in regard to stains used in the past and present to consider how histological stains should be improved. This triangulation helps to gather and assess in-depth data on past, present and future stain and staining techniques (Silverman, 2011).

## 4. Literature Review

### 4.1 Historical Histological Staining Techniques in Medicine and Biological Studies

The history of staining indicates that the application of histological techniques is a relatively new area of diseases diagnosis (Rodrigues et al., 2009). Historical staining techniques by early pathologists and surgeons were borrowed from a seventeen scientist Leeuwenhoek, who was instrumental in histology using substances such as Madder, indigo and saffron to stain tissues and using rudimentary microscopes to study them (Titford, 2009). These categories of early researchers used the microanatomy to draw a relationship among differences in cells as well as delineating a normal plant cell structure from that of the animal (Bancroft & Layton, 2013).

Later, newer techniques were devised to enhance the study of cell structure in detail using various laboratory chemicals to preserve tissues in their natural form before staining (Titford & Bowman, 2012). Joseph Von Gerlach was viewed as the pioneer of microscopical staining in 1858 when he used ammoniacal carmine successfully to stain cerebellum cells (Costa, Brito, Gomes, & Caliari, 2010).

The early histologists used the readily available chemicals to prepare tissues for microscopic studies; these laboratory chemicals were potassium dichromate, alcohol and the mercuric chloride to hard cellular tissues (Iyiola & Avwioro, 2011). These fixatives and staining agents were ingenious and after a period colored staining agents were developed which are still applicable in current laboratory staining techniques (Black, 2012). Examples of these ingenious colored stains still in use include the trichrome that is used in the liver and renal biopsies as well as the silver nitrate that is used in other organisms (Musumeci, 2014).

Great development in histologic stains was shaped by the improved technologic development of microscopes and the establishment of the histologic stains (aniline dye) in 1856 in Germany which manufacture a variety of new histological stains (Shostak, 2013). At the same time, research and knowledge relating to anatomy and tissues of the human body increased, and this knowledge was used to further research into new-histological techniques for the study of diseased tissue (Titford, 2009).

In the wake of the nineteenth century, many medical centers hired physicians, pathologists and surgeons to handle surgical issues (Titford & Bowman, 2012). It is this crop of pathologists who devised intraoperative staining techniques for frozen tissues sections by adapting a special staining technique in histopathology. It is during this time that the paraffin infiltration staining technique was devised (Shostak, 2013). Owing to this achievement, the non-malignant and the malignant tumors were studied, and a bacterium was identified as the causal organism of the disease in the nineteenth century (Godwin, 2011).

The Gram staining method was named after a Danish inventor Hans Christian Gram, who invented it as an approach to differentiating bacteria species in 1875 (Anderson, 2011). It is while working at the city morgue with his colleagues that Gram devised the technique of staining for the purpose of distinguishing the type of bacterium infection and also as a way of making the bacteria visible on selected and stained lung tissues during examination (Black, 2012). Although this technique was found unsuitable for certain bacterium organisms, it is still used today and competes fairly with modern molecular techniques of histology (Shostak, 2013).

### 4.2 Important Histological Stains Used in the Past and Present

**Carmine**

It is a commonly used stain in histology used by early botanists such as John Hill in their studies in 1770s (Jackson & Blythe, 2013). The stain was used to study microscopic tissue structures when in ammoniacal solution form and it is still used today in histologic studies. In particular, the stain was used widely by Rudolph Virchow (1821–1902) in microscopic studies; Virchow is considered as the ‘father of pathology’ (Musumeci, 2014).

**Hematin and Hematoxylin**

These are naturally occurring substances that have been in use in the history of histopathology (Titford, 2009). The stain was developed by Wilhelm von Waldeyer in 1863 and was obtained from a log tree found in Central America. Hematoxylin is a weak stain and is used with a combination of other solutions in oxidized form (Shostak, 2013).

In particular, the stain is combined with an oxidizer mordant to enhance its differentiating capacity of cell components; these solutions are called Hematoxylin. The versatility of the stain has enhanced the development of various Hematoxylin methods (Titford & Bowman, 2012). Historically, Hematoxylin was made into a nuclear stain that had shorter staining time and was resistant to acidic solutions; this made it suitable for histologic stain techniques requiring several steps (Anderson, 2011).

**Silver Nitrate**

Silver Nitrate has had a long usage in historical staining techniques and is still used in modern pathology. Initially, early researchers used silver nitrate to enhance the visibility of the tissue structure while studying it; this was done by applying solid silver nitrate on a tissue and then studying it (Titford & Bowman, 2012). The stain substance has been developed for many compounds, and confirmatory tests are needed when silver nitrate is used (Shostak, 2013). Silver nitrate stain has also been found to be reduced by argentaffin cells found in the epithelial linings of lungs, intestines, melanin and others (Musumeci, 2014).

However, methods have been devised to ‘tailor’ these tissues to avoid argyrophilic reactions when silver nitrate is used during staining process (Titford, 2009). In particular, methods such as the Gomori reticulin methods and the Grocott-Gomori method were devised to assess missing tissues and diseases in the liver and the rectum (Nadworny, Wang, Tredget, & Robert, 2010).

**Other Staining Procedures That Were Developed Recently**

**The Hematoxylin and Eosin Procedures**

Although historically used, there have been great laboratory changes in Hematoxylin stains; nearly all tissue specimens are treated with Hematoxylin and Eosin today (Bancroft & Layton, 2013). In addition, various Hematoxylin methods have been developed but all follow the same approach of staining tissue specimens in a hematoxylin, alcohol and tap or alkaline water to clear argentaffin agents. It has been found that most histopathological processes could be studied using the Hematoxylin and Eosin procedures (Titford & Bowman, 2012). In the same line, the method is quick to execute, cheap and can be altered. However, the Hematoxylin and Eosin are inefficient in that not all features of a substance can be received and special stains must be used (Musumeci, 2014).

**Romanowsky Stains–Giemsa Stains**

They were developed in the 1891 by Dimitri Romanowsky and popular for its multicolor in identifying blood parasites. The Giemsa Stains procedure is still used today. There has been great improvement in the stains, and its various methods make it applicable in paraffin-embedded, formalin-fixed and bone marrow biopsies (Musumeci, 2014).

**Gram Stain**

The Gram staining method was named after a Danish inventor Hans Christian Gram, who invented it as an approach to differentiating bacteria species in 1875 (Musumeci, 2014). Gram devised the technique of staining for the purpose of distinguishing the type of bacterium infection and also as a way of making the bacteria visible on selected and stained lung tissues during examination (Shostak, 2013). Although this technique was found unsuitable for certain bacterium organisms, it is still used today and competes fairly with modern molecular techniques of histology (Rudijanto, 2007). However, Gram technique is infallibly limited in the application on matters of environmental microbiology (Titford, 2009). That aside, Gram techniques had had success when performed on biopsy of infected parts and produced results quickly especially when there is a significant difference in prognosis and treatment. The method is often used in modern histology especially in paraffin fixatives for tissue sectioning (Titford & Bowman, 2012). In a recent case in Kuwait, the Gram staining technique was particularly effective in the diagnosis of Gonorrhea giving 99.4% effective results (Iyiola & Avwioro, 2011). The method is still used today especially with paraffin sections and has been modified to suit different substances.

**Trichrome Stains**

Historical assessment on the use of various stains in histology indicates that most pathologists were attracted by stains that gave multicolored results on the tissue specimens. As such, trichrome stains were developed from this need (Shostak, 2013). There have been various multiple stains such as blue–eosin, “triacid stain” by Ehrlich's (1888) and Masson's trichrome stain that has been popular in the modern histology. Trichrome stains show how complex the staining methods have become in the search of an efficient and consistent stain that would show fine, differentiated tissues (Musumeci, 2014).

### 4.3 Case Study Reviews

**Case Study 1**

This study was done in order to compare different staining methods and assess their effectiveness. The specific aim was to assess if the newly developed staining methods, the *Helicobacter pylori* silver stain HpSS methods and the modified McMullen's methods in the identification of H pylori organism. The method involved selecting tissue sections of gastric biopsies of 63 patients diagnosed with dyspepsia. The section tissues were stained using the four staining methods. In all the 63 cases, 30 sections tested positive for Helicobacter pylori while 30 tested negative for all cases of pylori infection while the remaining were tested using a combination of five histological tests (Anderson, 2011). The results indicated that, the interobserver stain method was the best for antibodies at 98%, followed by Giemsa at 87%, then the HpSS at 85%. At gold standard level, it was found that the Giemsa stain method was the best followed by McMullen's method (Rotimi, Cairns, Gray, Moayyedi, & Dixon, 2000). The study conclusions were that in all cases of staining, the *H pylori* infection was revealed; however, the modified Giemsa stain was the most effective for its sensitivity, ease of use, reproducibility and cost-effectiveness.

**Case Study 2**

The aim was to investigate the difference in capacity among different stains: Hematoxylin and Eosin, toluidine blue Stain, neuron-specific enolase (NSE) immunostaining and the S 100 protein. These stains were applied to assess the presence of neurons and mast cells in acute appendices Specimens were collected from clinically acute appendices categorized as histologically positive and negative. In the study all the 50 appendix specimens sections were subjected to Hematoxylin and Eosin, toluidine blue Stain, neuron-specific enolase (NSE) immunostaining and the S 100 protein. Hematoxylin and Eosin were applied as a routine stain for general study of the tissues while Toluidine blue stain was applied to enhance the easier study of mast cells. In addition, neuron-specific enolase (NSE) immunostaining was used as a marker and as well as the S 100 protein.

The results indicated that when comparing Hematoxylin and Eosin stain with S 100 they? showed 100% accuracy in identifying the denatured mucosal cells. However, the combination of these different staining methods resulted in a supplementary technique effective than the conventional staining method in observing changes and the pattern of diseased cells as well as the morphological shape of nerve fibers in the inflamed appendices (Russell & Gordon, 2009). In addition, the use of the several staining methods aided in confirming results of earlier stain diagnosis.

## 5. Results and Discussion

The literature review on staining techniques indicates that there has been great improvement in the histopathology and histotechnology. Historically, staining techniques used were carmine, silver nitrate, Giemsa, Trichrome Stains, Gram Stain and Hematoxylin among others (Titford & Bowman, 2012). These staining techniques are still in use although several modifications have been made to improve their efficiency. In other cases, some stain methods used earlier have been abandoned as they were toxic. Several staining techniques have been established to improve the staining methods.

There has been a rising need for efficient, accurate and less complex staining procedures (Harris & McCormick, 2010). The histopathology lab today is laden with a great work load and different types of histological assignments (Musumeci, 2014). As such, most histologists are more trained on special stains for particular works to give efficient results (Morelli, Porazzi, Ruspini, Restelli, & Banfi, 2013). In the history of histology, a great shift and development in histologic stains were shaped by improved technologic development of microscopes and the establishment of the histologic stains factory (aniline dye) in 1856 in Germany which manufactured variety of new-histological stains (Godwin, 2011).

These pathologists devised intraoperative staining techniques for frozen tissues sections by adapting a special staining technique in histopathology (Loreto, Leonardi, Musumeci, Pannone, & Castorina, 2013). It is during this time that the paraffin infiltration staining technique was devised (Titford, 2009). While these changes have taken place, there are old stain procedures that are still in use today and many others have been replaced with new immunal or staining techniques.

Additionally, the complexity of stains has been enhanced for the purpose of efficient and consistent staining processes that show fine and differentiated tissues (Ntziachristos, 2010).

## 6. Summary

Histological staining is a commonly used medical process in pathological diagnosis and forensic studies. The process of histological staining takes five key stages, and they include fixation, processing, embedding, sectioning and staining. Early histologists used the readily available chemicals to prepare tissues for microscopic studies; these laboratory chemicals were potassium dichromate, alcohol and the mercuric chloride to hard cellular tissues. These fixatives and staining agents were ingenious and after a period colored staining agents were developed which are still applicable in the laboratory staining techniques today.

Staining techniques used were; carmine, silver nitrate, Giemsa, Trichrome Stains, Gram Stain and Hematoxylin among others. There have been great changes in the techniques used for histological staining through chemical, molecular biology assays and immunological techniques collectively referred to us histochemistry and have facilitated greatly in the study of organs and tissues. Hematoxylin is a basic dye that is commonly used in this process and stains the nuclei giving it a bluish color while eosin (another stain dye used in histology) stains the cell's nucleus giving it a pinkish stain (Victor, 2013). While these changes have taken place, there are old stain procedures that are still in use today and many others have been replaced with new immunalstaining or staining techniques (Sine, 2014).

Some staining methods have been abandoned because the chemicals required have been medically proven to be toxic. Similarly, there have been great changes in workload requiring more advanced technics of staining. The case studies indicate that, in the modern histology a combination of different stain techniques are used to enhance the effectiveness of the staining process. In the modern histologic as a way of improving histological stains, several stains have been modified and combined with other stains to improve their effectiveness.
